# Recent advances in understanding hepatic drug transport

**DOI:** 10.12688/f1000research.9466.1

**Published:** 2016-10-06

**Authors:** Bruno Stieger, Bruno Hagenbuch

**Affiliations:** 1 Department of Clinical Pharmacology and Toxicology, University Hospital Zurich, University of Zurich, Zurich, 8091, Switzerland; 2 Department of Pharmacology, Toxicology and Therapeutics, The University of Kansas Medical Center, Kansas City, KS, 66160, USA

**Keywords:** drug disposition, drug metabolism, hepatocellular drug uptake, solute carrier

## Abstract

Cells need to strictly control their internal milieu, a function which is performed by the plasma membrane. Selective passage of molecules across the plasma membrane is controlled by transport proteins. As the liver is the central organ for drug metabolism, hepatocytes are equipped with numerous drug transporters expressed at the plasma membrane. Drug disposition includes absorption, distribution, metabolism, and elimination of a drug and hence multiple passages of drugs and their metabolites across membranes. Consequently, understanding the exact mechanisms of drug transporters is essential both in drug development and in drug therapy. While many drug transporters are expressed in hepatocytes, and some of them are well characterized, several transporters have only recently been identified as new drug transporters. Novel powerful tools to deorphanize (drug) transporters are being applied and show promising results. Although a large set of tools are available for studying transport *in vitro* and in isolated cells, tools for studying transport in living organisms, including humans, are evolving now and rely predominantly on imaging techniques, e.g. positron emission tomography. Imaging is an area which, certainly in the near future, will provide important insights into "transporters at work" *in vivo*.

## Role of drug transporters in drug disposition

Mammals, including humans, have evolved an elaborate array of organs, which are interconnected by the circulatory system. All organs are composed of cells, which can be considered as minimal functional units of organs. Cells need to strictly control their internal milieu and this is achieved by the plasma membrane, which acts as a barrier against the external milieu. The selective crossing of this barrier is mediated, among others, by transport proteins.

Drug disposition involves absorption, distribution, metabolism, and elimination of a drug. In order to enter the systemic circulation, orally administered drugs need to cross the epithelial barrier in the intestine and any drug in the systemic circulation needs to cross more than one cell membrane in order to be metabolized and/or eliminated ^1^. Given the barrier function of cell membranes, it is plausible that solutes including drugs and their metabolites need specific transport systems to cross this barrier in either direction ^2^. It should, however, be pointed out that this issue remains somewhat controversial ^3^. In addition, it is very likely that most, if not all, drug transporters are also involved in the handling of endogenous solutes and as such may serve in inter-organ communication ^1^.

The liver is the major site of drug metabolism and accounts for about 70% of drug elimination in humans ^4^. Consequently, this overview focuses on hepatocellular transporters. Overviews of hepatocellular drug transporters are found in ^4– 6^. From an overview perspective, drug transporters can be divided into two large super families: the solute carrier (SLC) superfamily mediates the cellular uptake of drugs in general, while members of the ATP-binding cassette (ABC) superfamily mediate the cellular efflux of drugs and their metabolites ^7^. As many of these transporters (in particular the SLC transporters involved in hepatocellular drug uptake, such as organic anion transporting polypeptides [OATPs], organic anion transporters [OATs], and organic cation transporters [OCTs]) are multi-specific, drug-drug interactions at these transporters may lead to altered pharmacokinetics and subsequently to adverse drug actions and/or to therapeutic failure ^8, 9^.

Consequently, there are numerous approaches used to develop models and tools for predicting drug-drug interactions and to incorporate this information into the process of drug development ^10^. While the crystal structures of human cytochrome P450 (CYP) enzymes were determined a while ago ^11^, such information on human drug transporters is lacking to date ^12^. This makes the *in vitro* prediction of pharmacokinetic drug-drug interactions challenging at best. Hence, currently, alternate approaches for studying drug-drug interactions are actively being pursued. One includes innovative cell culture approaches often termed "organ-on-a-chip" ^13, 14^.

Traditionally, drug-drug interaction studies early in drug development are performed *in vitro*. If, for example, an interaction at the cellular uptake level is to be considered, cell lines stably expressing drug transporters can be used. However, such experiments may be difficult owing to the complex kinetic properties of most drug transporters. For illustration, many OATPs transport their different substrates via different substrate-binding sites ^15^. Hence, testing for interactions of commonly used drugs with model substrates of OATP1B1 has yielded severalfold differences in the IC _50_ values for the same drug because different substrates were used in these tests ^16, 17^.

Once a new chemical entity (NCE) is clinically tested, the assessment of drug-drug interactions is a crucial part of such studies. Such studies are increasingly being supported by quantitative *in silico* predictions of altered pharmacokinetics of an NCE ^18^. Such approaches rely heavily on parameters determined *in vitro*. As an alternative method, imaging methodologies for studying drug (or solute) transport *in vivo* are rapidly emerging ^19– 21^. These approaches should also become useful for determining drug-drug interactions at the level of (uptake) transporters ^22^.

## Drug transporters and imaging

Imaging of the liver involves magnetic resonance imaging, positron emission tomography (PET), and scintigraphy and implies the use of labeled molecules ^23^. Dynamic imaging (i.e. with agents) of the liver may be aimed at obtaining insights into organ function, e.g. before performing major liver resections ^24^, or to study the uptake of solutes into the liver, e.g. for studying drug transport ^20^. PET studies have the advantage that they are performed under microdosing conditions and hence PET substrates are unlikely to exhibit pharmacological or even toxicological effects. In recent years, PET tracers for several drugs such as statins, metformin, telmisartan, or glyburide were developed and tested in animal models ^20^. (15 *R*)- ^11^C-TIC-Me was developed to visualize prostacyclin receptors by PET and was used in healthy volunteers for monitoring hepatobiliary transport ^22^. One of the major classes of solutes handled by the liver are the bile salts. Cholylsarcosine is a synthetic bile salt analogue ^25^ and can be modified to become a PET tracer ^26^. This makes the molecule an ideal tool for non-invasively studying transport processes in hepatocytes involving bile salt transporters. In pigs, [ *N*-methyl- ^11^C]cholylsarcosine was used to determine hepatobiliary secretion kinetics ^27^. [ *N*-methyl- ^11^C]cholylsarcosine was found to be concentrated 4000-fold from blood to bile. Importantly, no accumulation of cholylsarcosine was found in hepatocytes, demonstrating the concerted action of the basolateral uptake systems for bile salts, which in humans are represented by the sodium-taurocholate cotransporting polypeptide (NTCP), three OATPs, and the canalicular bile salt export pump BSEP ^28^. The canalicular efflux of cholylsarcosine was 180 times higher than the backflux from hepatocytes into the sinusoids, demonstrating the efficiency of transcellular bile salt flux under normal physiological conditions.

Transport proteins may have altered expression in various forms of liver diseases ^29^. Consequently, changes in the hepatocellular uptake of imaging tracers may indicate an altered expression of the respective uptake or efflux transporters. It should, however, be kept in mind that liver disease may also alter the energetics of hepatocytes, which in turn can affect driving forces for transporters and consequently impact the uptake of imaging tracers. For example, hepatobiliary scintigraphy can be used to assess the liver functional volume before liver resections ^30, 31^. Classically, indocyanine green clearance has been used for assessing dynamic liver function before liver surgery and has been reported to be superior to the Child-Pugh classification ^32^. One of the parameters used for Child-Pugh scoring is the total serum bilirubin level. It is known that bilirubin uptake into hepatocytes shares common (drug) transporters with indocyanine green ^33^, supporting the role of drug transporters in imaging.

## New drug transporters

As briefly outlined above, several members of the SLC families, in particular OATs and OCTs of the SLC22A and OATPs of the SLCO families, are well-characterized drug transporters ^34^. Some of these transporters are routinely included in drug-drug interaction tests for NCEs ^5, 35^. However, it is not yet known definitively whether additional transporters, especially transporters that have been characterized with respect to their normal physiological roles in maintaining solute movement across the hepatocyte membrane, are also involved in drug transport in and out of hepatocytes. For example, once characterized as the major hepatocellular uptake system for conjugated bile acids, NTCP was shown in 2006 to be able to transport rosuvastatin ^36^ and potentially account for up to 35% of rosuvastatin uptake in isolated human hepatocytes. In 2007, Shin and coworkers ^37^ identified and characterized a novel member of the SLC22A family, namely OAT7 ( *SLC22A9*), as a liver-specific OAT that is expressed at the sinusoidal membrane and was able to transport estrone-3-sulfate and dehydroepiandrosterone sulfate. In addition, transport of these sulfates was inhibited by several sulfated but not glucuronidated xenobiotics, and short-chain fatty acids were identified as potential physiological substrates of this transporter ^37^. Recently, in an attempt to better characterize the expression and function of OAT7, pravastatin was identified as the first drug substrate of OAT7 ^38^. It was previously shown that besides several OATPs ^34^, OAT3 is also able to transport pravastatin and rosuvastatin ^39^. Thus, we cannot rule out that additional transporters within a given family that have so far not demonstrated to be drug transporters could also be new drug transporters. Hence, new drug transporters may still be waiting to be discovered in hepatocytes.

## Drug transporters and regulatory guidance

Given the established role of transporters in drug disposition and pharmacokinetic drug-drug interactions, both the Food and Drug Administration (USA) and the European Medicines Agency as well as the Pharmaceuticals and Medical Devices Agency (Japan) have established guidelines on the investigation of the role of a specific set of transporters when developing NCEs ^40^. These guidelines require the determination of kinetic properties of transporters, e.g. the determination of IC _50_ values for NCEs. Unfortunately, the setup of transport assays will impact the numeric values of transporter parameters. This has been illustrated by a recent study, which used a common set of inhibitors for the multidrug resistance protein 1 ( *ABCB1*) in 23 laboratories for the determination of the IC _50_ values of these compounds. The outcome of this study is rather sobering in that the largest difference in IC _50_ values between the different laboratories for one inhibitor was 796-fold ^41^. As such, IC _50_ values are one of the parameters to be considered for the prediction of potential drug-drug interactions; aberrant IC _50_ values may lead to false negative or false positive predictions ^42^. The importance of proper experimental conditions has also been documented in a recent study where the effects of the unstirred water layer on the apparent K _m_ values for OCT2- and MATE1-mediated substrate transport were investigated ^43^. The authors concluded that increasing the expression levels of the expressed transporters in mammalian cells to increase the signal-to-noise ratio actually might lead to an overestimation of the apparent K _m_ values by 2- to 10-fold.

Such results clearly demonstrate a need for (some) harmonization of experimental conditions when kinetic parameters of transporter systems are being determined. Currently, such attempts are still at the beginning ^44– 46^. In addition to following standard rules for kinetic experiments, it is crucial for obtaining reproducible results to perform the transport experiments under conditions of initial linear uptake rates ^47^. If the latter condition is not fulfilled, estimated kinetic parameters may be invalid ^48^.

## Deorphanizing SLC transporters

Up to 10% of all human genes may have transport- or transporter-related functions ^7^. Currently, 456 SLC transporters, which belong to 52 subfamilies, are known in the human genome ^49^. Many of these SLC transporters are only marginally or not characterized at all. Although OATPs are normally called drug transporters, this notion is mainly because of the fact that the liver-expressed OATP1B1 and OATP1B3 are able to mediate the uptake of numerous drugs when expressed *in vitro* and polymorphisms in these two transporters are known to affect the pharmacokinetics of the drugs they transport. Besides these two best-characterized OATPs, there are nine additional genes in the human genome encoding OATPs and one pseudogene ^50^. Several of the encoded OATPs have been more or less well characterized and are known to be able to transport endobiotics as well as xenobiotics ^15, 50^. Among the better characterized are the multi-specific OATP1A2 and OATP2B1, and the transporters with a narrower substrate specificity like OATP1C1 or the prostaglandin transporter OATP2A1 ^34^. There are fewer reports characterizing the function of OATP3A1, OATP4A1, and OATP4C1, while for OATP5A1 and OATP6A1 no function has been reported so far and therefore OATP5A1 and OATP6A1 can be considered orphan transporters. Given that there are several reports that document OATP5A1 and OATP6A1 expression in cancer ^51– 53^, the elucidation of their function could be an important step towards better diagnosis or therapy for cancers expressing these OATPs. A recent study describes the biochemical characterization and expression of OATP5A1 in mature dendritic cells and whereby OATP5A1 seems to affect cell proliferation ^54^. However, no transport function was identified, and as long as no other biological function is associated with OATP5A1, it still needs to be deorphanized.

Similar to the OATP family with two orphan transporters, there are several orphan transporters in the SLC10A family that contain the hepatocellular sodium-dependent bile acid uptake transporter NTCP ( *SLC10A1*), the apical sodium-dependent bile acid transporter ASBT ( *SLC10A2*) expressed in the ileum, kidney, and cholangiocytes, and the sodium-dependent organic anion transporter SOAT ( *SLC10A6*) ^55^. While these three transporters have been well characterized, this family contains four additional proteins, namely P3 ( *SLC10A3*), P4 ( *SLC10A4*), P5 ( *SLC10A5*), and P7 ( *SLC10A7*), with mostly unknown function. To try to understand the function of SLC10A4 (a "transporter" expressed in humans in the developing ventral mesencephalon ^56^ and in rats in cholinergic and monoaminergic neurons ^57^), mice lacking SLC10A4 protein were recently characterized ^58– 60^. No direct transporter function could be demonstrated for SLC10A4, but its absence reduced dopamine, noradrenaline, serotonin, and acetylcholine content in certain brain regions. These knockout mice also showed characteristics that are similar to symptoms of neurodegenerative disease and, as a consequence, SLC10A4 might become a novel target for neurological and mental diseases. An additional example is SLC32F2, which was known to be highly expressed in tumors ^61^, but only recently it was demonstrated that this transporter may mediate cellular uptake of the anticancer drug YM155 ^62^. Furthermore, SLC38A9 was only recently identified as an important component of the lysosomal amino acid sensing machinery and to be involved in the control of the mammalian target of rapamycin complex 1 ^63^. These few examples highlight the pressing need for "deorphanizing" the SLC transporters completely. Possible methods may include the generation of knockout mice as described for SLC10A4 ^58, 60^, the use of haploid genetic screens, which was successful in the identification of a function for SLC35F2 ^62^, and possibly siRNA screens when a transporter for a given function needs to be identified.

## Outlook

There is clearly a need for obtaining more information on the role of SLC transporters in drug transport as well as for deorphanizing SLC transporters ^49^. The identification of substrates for transporters may not be straightforward, e.g. SLC10A4 is, to the best of our knowledge, a transporter whose substrates have not been identified conclusively yet. While one group reported that protease activation of SLC10A4 makes this protein a bile acid transporter ^64^, another group could not reproduce this finding and also did not find evidence for a transport activity of neuromodulators ^65^. One possible explanation for these different findings is the use of different experimental systems. Alternatively, the fact that NTCP was found to form homodimers and heterodimers ^66^ might indicate that SLC10A4 may need an interacting protein to function properly. So far, very limited information on the impact of homodimer and heterodimer formation on transport activity and substrate specificity of transport proteins is available. With the development of very powerful tools for the determination of protein interactomes ^67^ including membrane proteins ^68^, a non-targeted search for interacting partners for orphan transporters might become a feasible option in the near future. In support of this, the determination of the interactome of yeast ABC transporters has yielded a wealth of information for the physiological role and regulation of these transporters ^69^. Rat multidrug-resistance-associated protein 6 (MRP6) ( *Abcc6*) was cloned in 2000 and BQ123 was identified as the only substrate ^70^. In the same year, mutations in *ABCC6* were found to cause pseudoxanthoma elasticum ^71, 72^. Human *ABCC6* was found to transport glutathione conjugates and leukotrienes ^73^. However, a physiological substrate for MRP6 remained enigmatic ^74^. An elegant metabolomics approach using vesicles derived from HEK293 cells overexpressing MRP6 led to the identification of ATP as a potential physiological substrate for MRP6 ^75, 76^. This finding demonstrates the power of metabolomics in identifying substrates for drug and orphan transporters.

The channeling of substrates for energy production through complexes of sequential metabolic enzymes has been known for quite some time ^77, 78^. The role of transporter-metabolizing enzyme interactions in pharmacokinetics as well as in drug-drug interactions is starting to gain attention ^18, 79^. The clinical relevance of this approach is illustrated by a study in which rifampicin was tested as an OATP inhibitor as well as an inducer of CYP3A4. As an OATP inhibitor, rifampicin increased the trough concentration for the victim drug bosentan, while as an inducer of CYP3A4 it decreased the AUC for bosentan ^80^.

Information on transporter expression levels is an important prerequisite for the prediction of pharmacokinetics and drug-drug interactions occurring at hepatocellular transporters ^81^. Absolute quantification of transporters by proteomics resulted in first data ^82, 83^. From the data published so far, it seems that under normal physiological conditions a considerable interindividual variability of transporter levels exists. This variability often exceeds a 10-fold range. To what extent technical issues, e.g. the condition of liver biopsies used for transporter quantification, contribute to this observation remains to be determined. Furthermore, it should be kept in mind that many forms of (liver) disease have an impact on transporter expression levels ^29, 84^.

## Abbreviations

ABC, ATP-binding cassette; CYP, cytochrome P450; MRP, multidrug resistance-associated protein; NCE, new chemical entity; OAT, organic anion transporter; OATP, organic anion transporting polypeptide; OCT, organic cation transporter; PET, positron emission tomography; SLC, solute carrier.
